# NTCP gene polymorphisms and hepatitis B virus infection status in a Ghanaian population

**DOI:** 10.1186/s12985-020-01376-0

**Published:** 2020-07-03

**Authors:** Eric Nyarko, Christian Obirikorang, W. K. B. A. Owiredu, Evans Asamoah Adu, Emmanuel Acheampong, Freeman Aidoo, Emmanuel Ofori, Bright Selorm Addy, Henry Asare-Anane

**Affiliations:** 1 Department of Molecular Medicine, School of Medicine and Dentistry|, Kwame Nkrumah University of Science and Technology, Kumasi, Ghana; 2 Department of Chemical Pathology, University of Ghana Medical School, Accra, Ghana; 3 School of Pharmacy, Central University, Accra, Ghana; 4 School of Medical and Health Science, Edith Cowan University, Joondalup, Australia

**Keywords:** SLC10A1, NTCP, SNP, CHB infection, PCR-RFLP

## Abstract

**Background:**

SLC10A1 gene codes NTCP, a receptor through which the hepatitis B virus (HBV) gets access into hepatocytes - a stage of the viral cycle necessary for replication. Polymorphism variants of SLC10A1 play roles in HBV infection, viral clearance, treatment outcome, and complications, in diverse ethnic groups and countries. However, no such study has been conducted in the Ghanaian population, a country with HBV endemicity. Therefore, an exploratory study was conducted to investigate the presence of three (3) single nucleotide polymorphisms (SNPs) in the SLC10A1 gene (rs2296651, rs61745930, and rs4646287) and assessed the risk of HBV infection among the Ghanaian population.

**Method:**

Polymerase chain reaction-restriction fragment length polymorphism (PCR-RFLP) method was used to determine the presence of the SNPs among 292 participants comprising 146 HBV infected persons as case-subjects and 146 HBV non-infected persons as control-subjects.

**Results:**

The minor allele frequency (T) of rs2296651 was present in a significantly high proportion of cases compared with the control group (11.6% vs. 3.1%, *p* < 0.0001). The homozygote recessive variant of rs61745930 was present in 2.7% of the control group and 5.5% of the case group. Moreover, the minor allele frequencies of rs4646287 were 9.3 and 8.2% among the control and the case group, respectively (*p* = 0.767). Under the dominant (CC) genetic model of inheritance, rs2296651 was found to be protective of HBV infection [OR = 0.18 (0.07–0.44)], whereas under the co-dominant and additive model, rs2296651 was a potential risk factor for HBV infection [OR = 5.2 (95%CI: 2.1–12.8); 3.5 (95%CI: 1.6–7.6], respectively. Variants of rs61745930 and rs4646287 were not associated with HBV infection (*p* > 0.05). Polymorphisms in SLC10A1, however, did not show any significant association with HBV infectivity (*p* > 0.05).

**Conclusion:**

The study highlights some polymorphism proof that variants rs2296651, rs61745930, and rs4646287 exist in HBV-infected individuals in Ghana. Although variant rs2296651 was found to be associated with HBV infection, this association warrants more studies. Polymorphisms in SLC10A1 were not associated with HBV infectivity among the Ghanaian population. Further investigation is warranted to assess the offensive role of the relationship between rs2296651 and HBV infectivity.

## Introduction

The prevalence of hepatitis B virus (HBV) infection in regions of high endemicity ranges from 6 to 8%; in Africa, prevalence rates of 10–15% have been reported [1, 2]. Within the adult population in Ghana, prevalence rates between 10 and 13% have been recorded across the ten regions with a national average rate of 12.3% [3], which is about 50% higher than the current global average. This predisposes at least 1/10th of every Ghanaian adult to the risk of later suffering or even dying from HBV- associated complications such as cirrhosis or Hepatocellular carcinoma (HCC) [4, 5]. HBV infection, progression, and complications are affected by factors such as genes, race, gender, age, and expression of some biomolecules such as CD4^+^ T-helper cells [6]. Spontaneous clearance of HBV infection is associated with vigorous polyclonal and multi-specific CD4^+^T-helper cell response, compared to the weak responses observed in persistent or chronic infections [7]. Solute carrier family 10 A1 (SLC10A1) gene-coded Na^+^-taurocholate co-transporting polypeptide (NTCP) is another molecule that influences the outcome of HBV infection. It is a protein receptor through which the HBV gets access into the hepatocytes, a stage of the viral cycle necessary for replication [8, 9]. Thus, NTCP could represent a molecular and pharmacologic target for treatment and prevention.

With the high risk of HBV infection and its associated end-stage liver complications, the lack of a cure for HBV infection, high treatment cost [10, 11], low long term benefits and high toxicity associated with interferon, nucleoside and nucleotide therapies; new treatments and methods targeting different molecules are warranted. Single nucleotide polymorphic (SNP) variants of the NTCP gene – SLC10A1 such as rs7154439, rs4646287 [4, 12, 13], rs2296651 and rs61745930 [14–16] is associated with HBV persistent infection (chronicity), expression of HBeAg and viral clearance among different ethnic groups and countries. The polymorphic SNP variants in the Ghanaian population or responsible for any of the HBV infection status are non-existent. Therefore, this explorative study was conducted to determine the presence three (3) SNPs in the SLC10A1 gene (rs2296651, rs61745930, and rs4646287) and the risk of HBV infection among the Ghanaian population.

## Methods

### Study design and participant selection

This was a case-control study. Two hundred and ninety-two (292) participants comprising 146 HBV infected persons as cases (excluding active hepatitis B cases) and 146 apparent healthy HBV non-infected persons as controls were recruited from the Greater Accra Region of Ghana All the participants selected were Ghanaians by birth between 18 and 65 years of age.

### Sample size justification

The sample size for the study was calculated using the Cochran–Armitage trend tests [17]. By considering the additive genetic model at a case: control ratios of 1:1, the rare allele frequency (0.079) of the most replicated SNP (rs2296651), and a national HBV average prevalence of 12.3% [3], the sample size needed to achieve the prespecified 0.05 α-level and a power 80 for a two-sided trend test was 348 (174 cases vs 174 controls). The total response rate of the participants was approximately 84.0% (292/348). The final population of the study constituted 292 participants comprising of 146 cases and 146 controls.

### Sample collection, preparation, biochemical analysis, and DNA isolation

Nine (9) millilitres of venous blood was drawn from each participant into a 3 ml serum separator gel tube (SST) and two 3 ml EDTA tubes; thus, one for haematology and the other for DNA extraction. This was done to avoid contamination. The SST sample was allowed to clot and centrifuged at 2500 g for 7mins to obtain serum and preserved between − 16 to − 20 °C till assayed. Mindray 5-parts hematology analyzer (Mindray, Shenzhen-China, 2013) was used to quantify blood hemoglobin, blood cells and other cellular indices one EDTA sample, using the impedance, flow cytometry, and vis -spectrophotometry methods depending on the analyte or index (Mindray, 2013, 2018). HumaStar 200 Clinical chemistry analyzer (Human Diagnostics worldwide, Germany, 2014) was used to quantify the concentrations of total and direct bilirubin, total protein, albumin; and the activity of the enzymes: alanine aminotransferase (ALT), aspartate aminotransferase (AST), gamma-glutamyl transferase (ϒGT) and alkaline phosphatase (ALP) (Human Diagnostics worldwide, Germany, 2014, 2018). Genomic (g) DNA was isolated from the other EDTA anticoagulated sample as described by Suguna, et al. [18], and preserved below − 80 °C until genotyped.

### Profiling of HBV antigens and HBV -host antibodies

The commercial Clinogen HBV-5 card was used to screen for the presence of two (2) HBV viral antigens and three (3) host antibodies using colloidal gold and membrane chromatography technology (Clinogen Diagnostics, Japan) after ‘running’ HBV profile on ELISA-confirmed controls previously obtained from the Noguchi Memorial Institute for Medical Research (NMIMR), Legon, Accra. HBsAg, HBeAg, and HBsAb were screened for with the Immunochromatographic (dual-antibody sandwich) principle, and the HBeAb and HBcAb were screened for by the neutralization competitive inhibition principle (Clinogen Diagnostics, Japan; Clinogen Diagnostics, UK).

### Primer design

Primers spanning each of the SNP variants (rs2296651, rs61745930, and rs4646287) were designed using the NCBI Primer-BLAST software and the results shown in Table S1 (https://www.ncbi.nlm.nih.gov/tools/primer-blast/index.cgi). Table S1 shows PCR primers and their characteristics restriction enzymes.

### Genotyping

#### PCR conditions

To detect the presence of the rs2296651 (about 200 bp, 80), rs61745930 (about 400 bp, 250 bp), and rs4646287 (about 140 bp, 250) fragments, a 25 μL PCR reaction volume consisting of 8.5 μL nuclease-free water, 1 μL (10 μM) each of forward and reverse primers, 12.5 μL of One Taq 2x PCR Master mix (New England BioLabs (NEB), USA) and 2 μL of the isolated genomic DNA was prepared. Nuclease free water was used in place of the gDNA in the negative control. PCR amplification was done using the Applied Biosystems thermal cycler (Fisher Scientific, USA). Denaturation was completed at 94^0^ C for 3 mins (initial) and 94^0^ C for 30 s (on rest). Annealing conditions for rs2296651, rs61745930 and rs4646287 were 58^0^ C (30 s), 59.5^0^ C (35 s) and 60.0^0^ C (30 s), respectively. Initial and final extension were completed at 72^0^ C (30 s) and 72^0^ C (10 mins), respectively. The denaturation, annealing and extension steps were done for 35 cycles, and the hold after the final extension was at 4 °C.

#### Restriction digestion

The optimal enzyme concentration was prepared with the appropriate assay buffer containing 1 μg of substrate DNA according to Manufacturer’s instructions. Controls were included to ensure there was no contamination or unspecific product obtained. To complete the genotyping of rs2296651, 15 μL of the PCR product was added to 0.2 μL of *HphI* enzyme (NEB, USA) and dissolved in 1.5 μL of NEB buffer and 3.3 μL of nuclease-free water, and incubated at 37 ^0^ C for 11 min for the enzyme digestion of the PCR product. Similarly, 15 μL of the PCR product was either added to 0.2 μL of *BsaBI* or 0.2 μL of *Taq*^*ἀ*^*I* enzymes (NEB, USA) and dissolved in 1.5 μL of NEB buffer and 3.3 μL of nuclease-free water and incubated at 60 ^0^ C for 60 min (1 h) for the enzyme digestion of either rs61745930 or rs4646287.

#### Product visualization

The digested fragments were separated with a 2.5% EtBr- incorporated agarose gel at 100 V, 2A for 90 min, and using the Quick-Load purple 100 bp DNA Ladder (NEB, USA) as the molecular marker (MM) and visualized under UV trans-illuminator. The genotypes were determined according to the band patterns/sizes and in comparison, to the molecular marker (MM) as shown in the supplementary figures. Figure S1, Figure S2 and Figure S3 show the gel bands for the SNP rs2296651, rs61745930, and rs4646287, respectively.

### Statistical analysis

Results obtained were entered into the Statistical Package for the Social Sciences (SPSS), coded, and analyzed using this SPSS (version 23.0). Frequencies were used to represent categorical data and compared using Chi-Square test analysis to compare the genotype and allele frequencies between the groups. Skewed data were compared using the Man-Whitney Test. Normally distributed data were represented with mean ± standard deviation and compared between groups using the T-test. To test for associations between HBV and every single SNP, logistic regression models were fitted, in which each SNP was presented as a predictor variable whose values were equal to the number of copies of the minor allele (0, 1, 2) in an additive model, or presence of at least one copy of the minor allele (0, 1) in a dominant model or presence of two copies of the minor allele (0, 1) in a recessive model. Sex, age and family history of HBV status were included as covariates in the fitted model. The structure of the model was represented as: Logit *[pr(D = 1)] = α + β 1 G.*

Where D denotes HBV phenotype; G denotes SNP coded as an additive, dominant or recessive; β denotes the corresponding coefficient for each variable in the model, and its exponential is the corresponding odds ratio. A *P*-value of less than 0.05 was considered statistically significant.

## Results

Table 1 shows the comparison of age, gender, biochemical indices between cases and controls. The male and female participants were equivalently distributed among cases and controls (*p* = 0.193). Total protein, direct bilirubin, ALT, ALP and de-Ritis ratio did not show any significant differences between cases and control participants (*p* > 0.05). However, total bilirubin (*p* = 0.002), indirect bilirubin (*p* < 0.0001), and AST (*p* = 0.007) were significantly higher in cases compared with controls. On the other hand, albumin (*p* = 0.010) and GGT (*p* = 0.042) were significantly higher in controls compared with cases. The aspartate aminotransferase-to-platelet ratio index (APRI index) (*p* = 0.003), fibrosis index based on four factors (FIB-4) (*p* = 0.009) and RDW-to-platelet ratio (RPR) (*p* < 0.0001) were significantly higher in the case-subjects compared to control subjects. Table S2 shows the measured hematological parameters among cases and control subjects. Apart from RBC count, MCV, PCT, and hematocrit level (*p* > 0.05), cases and control participants significantly differed in terms of measured hematological parameters.

**Table 1:** Descriptive summary of demographic and liver function indices of the study participants

Variables	Control *n* = 146	Cases *n* = 146	*P*-value
**Gender**^**a**^			0.193
Male	68 (46.6)	57 (39.0)	
Female	78 (53.4)	89 (61.0)	
**Age (years)**	31.3 ± 10.0	30.6 ± 8.1	0.542
**Total Bilirubin (**μmol**/L)**	12.73 ± 5.29	14.89 ± 6.56	**0.002**
**Direct Bilirubin (μmol/L)**	6.59 ± 3.54	6.44 ± 3.22	0.700
**Indirect Bilirubin (μmol/L)**	6.08 ± 3.27	8.13 ± 4.50	**< 0.0001**
**Total protein (g/L)**	72.35 ± 7.52	70.83 ± 9.97	0.141
**Albumin (g/L)**	41.69 ± 5.53	39.72 ± 7.35	**0.010**
**ALT (U/L**	26.42 ± 11.79	27.33 ± 13.59	0.540
**AST (U/L)**	27.14 ± 13.30	30.35 ± 19.10	**0.007**
**GGT (U/L)**	33.37 ± 16.99	27.72 ± 12.73	**0.042**
**ALP (U/L)**	159.7 ± 55.8	159.0 ± 53.	0.914
**De Ritis ratio**	1.0 (0.80–1.26)	1.03 (0.76–1.47)	0.631
**APRI index**	0.30 (0.20–0.43)	0.37 (0.25–0.50)	**0.003**
**FIB-4**	0.68 (0.49–1.05)	0.80 (0.60–1.18)	**0.009**
**RPR**	0.06 (0.04–0.07)	0.07 (0.06–0.10)	**< 0.0001**

Data is presented as means ± standard deviation. Variables with the superscript “a” were presented as frequency and subgroup proportions. *ALT* Alanine aminotransferase, *AST* Aspartate aminotransferase, *GGT* Gamma-glutamyl transferase, *APRI index* Aspartate aminotransferase-to-platelet ratio index, *FIB-4* fibrosis index based on four factors, *RPR* RDW-to-platelet ratio, *P* < 0.05 is considered statistically significant

The genotype and allele frequencies of SLC10A1 gene polymorphism were compared between cases and control participants (Table 2). The minor allele frequency (T) of rs2296651 was presenting in a significantly high proportion of cases compared with the control group (0.116 vs 0.031, *p* < 0.0001). Also, the heterozygote (18.5% vs 6.2%, *p* = 0.001) and homozygote recessive (3.4% vs 0%, *p* = 0.025) variants of rs2296651 were present in significantly higher proportions of the case group than the control group, respectively. The homozygote recessive variant of rs61745930 was present in 2.7% of the control group and 5.5% of the case group. Moreover, the minor allele frequencies of rs4646287 were 9.3 and 8.2% among the control and the case group, respectively (*p* = 0.767) (Table 2).

**Table 2:** Genotype, allele frequencies of SLC10A1 gene polymorphism in the study participants

SNP	Controls (*n* = 146)			Cases (*n* = 146)			^†^*P*-value
	n (%)	Allele frequency		n (%)	Allele frequency		
		C	T		C	T	
**rs2296651**		0.969	**0.031**		0.884	**0.116**	**< 0.0001**
CC	137 (93.8)			114 (78.1)			**0.0001**
CT	9 (6.2)			27 (18.5)			**0.001**
TT	0^a^			5 (3.4)			**0.025**
*HWE-P*^*‡*^	1.00			0.112			
**rs61745930**		**0.014**	0.986		**0.028**	0.972	0.238
TT	142 (97.3)			138 (94.5)			0.228
CT	0			0			–
CC	4 (2.7)			8 (5.5)			0.228
*HWE-P*^*‡*^	**0.003**			**0.009**			0.888
**rs4646287**		0.907	**0.093**		0.918	**0.082**	0.767
CC	120 (82.2)			123 (84.2)			0.648
CT	24 (16.4)			21 (14.4)			0.636
TT	2 (1.4)			2 (1.4)			1.00
*HWE-P*^*‡*^	0.763			0.549			

Cases: HBV seropositive, Controls: HBV seronegative, *HWE-P* Hardy-Weinberg equation *p*-values, ^†^*P*-values represents the chi-square test to compare genotype frequency between case-and control -subjects. *P*-values for chi-squared test for variant allelic frequency based on HWE, *p* < 0.05 depicts it not consistent with HWE

Table S3 shows the re-categorised into HBeAg positive (active) and HBeAg negative (inactive) based on HBV antigen/antibody profiling. The results indicated that 39.0% were active CHB cases whereas 61.0% were inactive CHB cases.

The genotype and allele frequencies of SLC10A1 gene polymorphism in active CHB and inactive CHB cases are shown in Table 3. There were no significant differences in allele and genotype frequencies among HBeAg positive and HBeAg negative cases (*p* > 0.05) Table 3.

**Table 3:** Genotype, allele frequencies of SLC10A1 gene polymorphism in active and inactive cases

SNP	Inactive CHB cases (*n* = 89)			Active CHB cases (*n* = 57)			^†^*P*-value
	N (%)	Allele frequency		N (%)	Allele frequency		
		C	T		C	T	
**rs2296651**		0.882	0.118		0.860	0.140	0.240
CC	72 (80.9)			42 (73.7)			
CT	13 (14.6)			14 (24.6)			
TT	4 (4.5)			1 (1.8)			
*HWE-P*^*‡*^	0.123			0.112			
**rs61745930**		**0.044**	0.956		**0.035**	0.965	0.382
TT	85 (95.5)			53 (93.0)			
CT	0			0			
CC	4 (4.5)			4 (7.0)			
*HWE-P*^*‡*^		**0.013**			**0.009**		
**rs4646287**		0.916	0.084		0.912	0.088	0.374
CC	76 (85.4)			47 (82.5)			
CT	11 (12.4)			10 (17.5)			
TT	2 (2.2)			0			
*HWE-P*^*‡*^	0.615			0.821			

*HWE-P* Hardy-Weinberg equation p-values, ^†^P-values represents the chi-square test to compare genotype frequency between case-and control -subjects. *P*-values for chi-squared test for variant allelic frequency based on HWE, *p* < 0.05 depicts it not consistent with HWE

The association between SLC10A1 variants and HBV infection was compared under the recessive, dominant, co-dominant and additive model of inheritance. Under the dominant model, rs2296651 was found to be protective of HBV infection, whereas, under the co-dominant and additive model, rs2296651 was a risk factor for HBV infection (Table 4). Polymorphisms in rs61745930 and rs4646287 were not significantly associated with HBV infection under any of the models (Table 4).

**Table 4:** Odds ratios and *P*-values of SLC10A1 gene polymorphism under recessive, dominant, co-dominant and additive models of inheritance

Model	Alleles	aOR (95% CI)	bOR (95% CI)
**rs2296651**			
Recessive Model	mt/wt CT + wt/wt TT	1 (reference)	1 (reference)
	mt/mt CC	n/a	2.70 (0.29–24.78)
Dominant Model	mt/wt TC + mt/mt TT	1 (reference)	1 (reference)
	wt/wt CC	0.18 (0.07–0.44) *	1.55 (0.70–3.43)
Co-dominant model	wt/wt CC + mt/mt TT	1 (reference)	1 (reference)
	mt/wt TC	5.2 (2.1–12.8) *	0.85 (0.4–1.6)
Additive model 1	mt/wt TC	1 (reference)	1 (reference)
	mt/mt TT	5.23 (2.09–13.11) *	0.49 (0.20–1.16)
**rs61745930**			
Additive Model	wt/wt TT	1 (reference)	1 (reference)
	mt/mt CC	1.5 (0.7–3.2)	0.80 (0.4–1.6)
**rs4646287**			
Recessive Model	mt/wt TC + wt/wt TT	1 (reference)	1 (reference)
	mt/mt TT	n/a	n/a
Dominant Model	mt/wt TC + mt/mt TT	1 (reference)	1 (reference)
	wt/wt CC	0.81 (0.35–1.85)	1.31 (0.53–3.24)
Co-dominant model	wt/wt CC + mt/mt TT	1 (reference)	1 (reference)
	mt/wt CT	1.10 (0.50–2.4)	0.96 (0.4–2.2)
Additive model 1	mt/wt TC	1 (reference)	1 (reference)
	mt/mt TT	1.34 (0.58–3.10)	0.65 (0.25–1.66)

*mt* mutant type, *wt* wild type, Highlighted values represent statistically significant values. CI-confidence interval; “*” represent statistically significant model, p f < 0.05. aOR- odds ratios for controls vs cases; bOR- odds ratios HBeAg positive and HBeAg negative

## Discussion

Several studies have reported the presence of SLC10A1 rs2296651, rs61745930, and rs4646287 SNP variants among persons with HBV infection (4, 14, 24). Thus, we determined the association between 3 SNPs in the SLC10A1 gene (rs2296651, rs61745930, and rs4646287) and the risk of HBV infection among the Ghanaian population. Our results indicated that the MAF of rs2296651 was 3.1% among HBV uninfected population and 11.6% among HBV infected population. These findings are similar to those reported among Korean (3.1%) and Chinese-Americans (7.5%) populations [14]. Also, similar findings have been replicated among Chinese and Vietnamese in a study by Wang*,* et al. [13]. Moreover, Li*,* et al. [15] reported a 4.4% MAF of rs2296651 among the Chinese Han population. In a more recent study among Vietnamese, the rs2296651 variant was reported in an average of 6% of the population, with a T minor allele frequency of 3% [19].

Also, the distribution of the MAF of rs61745930 and rs4646287 were 1.4 and 9.3% among HBV uninfected population, and 2.8 and 8.2% among HBV infected population. Variants of rs4646287 had the highest frequency among the study participants, with about 16.4 and 1.4% of the Ghanaian HBV uninfected population carrying the codominant (CT) and recessive (TT) variants, respectively. Lower frequencies of the variant than these were observed among the Chinese Han [20] and Chinese [21] population. Also, Chuaypen*,* et al. [22] reported rs4646287 average MAF of 17.3% among the Thai population with CT and TT genotype frequencies of 16 and 1.3% respectively and the T minor allele was found to be 9.3%. These findings indicate that the rs2296651, rs61745930, and rs4646287 SLC10A1 polymorphisms -its genotypes and alleles- are widely distributed among Asians and the Ghanaian population. However, some studies have reported the absence or low prevalence of these SLC10A1 SNP variants in Caucasians [23]. Thus, replication studies among a wider population of Ghanaians and the entire of Africa are warranted since these countries represent HBV endemic territories.

We observed that variants of rs2296651 were associated with the risk of HBV infection. However, the association was independent of HBV infectivity. Under the recessive, additive and codominant model of inheritance, we observed that rs2296651 was associated with the increased risk of HBV infection. On the other hand, under the dominant genetic inheritance model, we observed that rs2296651 was protective of HBV infection. Several studies have indicated that variants of rs2296651 either increases an individual’s susceptibility to HBV infection and/or its complications, makes resolution/viral clearance of the infection faster or improves the prognostic outcome of cirrhosis and HCC [15, 19–22]. The evidence of the transferability of rs2296651 associated with HBV infection has been demonstrated among the Han population [15], Taiwanese [24] and Vietnamese [19]. However, unlike our results, rs2296651 [(c.800C > T) (S267F)] has been associated with reduced HBV infection and associated hepatic complications [25]. Among the population of Thailand, the rs2296651 SNP variant was associated with inactive and non-infectious CHB and viral clearance among HBV infected persons [22]. These findings indicate that although variants of rs2296651 are associated with HBV infection, the definition of the risk variant may be dependent on the genetic architecture of the population as well as the environmental agents identified as risk factors for HBV infection.

The study has some limitations that should be acknowledged. The sample of the study was small which may affect power replication of the odds of NTCP polymorphic variants associated with HBV infection. Also, the study was localized, and generalization of findings should be done with caution, and therefore recommend replication studies in other parts of the country considering the limitation of this study. Although further studies are required, we have demonstrated the feasibility of the association of rs2296651 with HBV infection in the Ghanaian population and our findings are congruent with previous reports among the Asian populations.

## Conclusion

The study highlights some polymorphism proof that variants of rs2296651, rs61745930, and rs4646287 exist in HBV-infected individuals in Ghana. Although, variant rs2296651 was found to be associated with HBV infection. Polymorphisms in SLC10A1 is not associated with HBV infectivity among the Ghanaian population. Further investigation is warranted to assess the predisposition role of the relationship between rs2296651 and HBV infectivity.

## Supplementary information

**Additional file 1: Table S1.** PCR primers and their characteristics restriction enzymes. Table S1 shows the primers spanning each of the SNP variants (rs2296651, rs61745930, and rs4646287) were designed using the NCBI Primer-BLAST software. **Table S2.** Hematological profile of the study participants. Table S2 shows the measured hematological parameters among cases and control subjects. **Table S3.** Antigens and host antibodies profiling of HBV positive cases. Table S3 shows the re-categorised into HBeAg positive (active) and HBeAg negative (inactive) based on HBV antigen/antibody profiling.

**Additional file 2: Figure S1.** Gel bands for the SNP rs2296651 obtained using the PCR-RFLP. Figure S1 shows the gel bands for the SNP rs2296651 obtained using the PCR-RFLP. MM = the molecular marker or ladder; NC = the negative control; C (200 bp), T (120 bp). Samples 51–60 are cases and samples 291–300 are controls.

**Additional file 3: Figure S2.** Gel bands for the SNP rs61745930 obtained using the PCR-RFLP. Figure S2 shows the gel bands for the SNP rs61745930 obtained using the PCR-RFLP. MM = the molecular marker or ladder; NC = the negative control; C (400 bp), T (250). Ca = Cases and Co = Controls. Samples 22–25, 27, 30 and 33 are cases and samples 243, 249,252, 318, 320–322 are controls.

**Additional file 4: Figure S3.** Gel bands for the SNP rs4646287 obtained using the PCR-RFLP. Figure S3 shows the gel bands for the SNP rs4646287 obtained using the PCR-RFLP. MM = the molecular marker or ladder; C (240 bp), T (140). Samples 35–46 are cases; and samples 201–205 plus 286–290 are controls.

## Data Availability

The datasets used and/or analysed during the current study are available from the corresponding author on reasonable request.

## Abbreviations

HBV: Hepatitis B virus
MAF: Minor allele frequency
SNP: Single nucleotide polymorphism
SLC10A1: Solute carrier family 10 A1
NTCP: Na^+^-taurocholate co-transporting polypeptide
HCC: Hepatocellular carcinoma
PCR-RFLP: Polymerase chain reaction-restriction fragment length polymorphism
