# Supplementary material for: NTCP gene polymorphisms and hepatitis B virus infection status in a Ghanaian population
Source: Virol J. 2020 Jul 3;17:91. doi: 10.1186/s12985-020-01376-0 (PMC7333392; doi:10.1186/s12985-020-01376-0)
Supplement: Supplementary file 1 — Additional file 1: Table S1. PCR primers and their characteristics restriction enzymes. Table S1 shows the primers spanning each of the SNP variants (rs2296651, rs61745930, and rs4646287) were designed using the NCBI Primer-BLAST software. Table S2. Hematological profile of the study participants. Table S2 shows the measured hematological parameters among cases and control subjects. Table S3. Antigens and host antibodies profiling of HBV positive cases. Table S3 shows the re-categorised into HBeAg positive (active) and HBeAg negative (inactive) based on HBV antigen/antibody profiling. [file 12985_2020_1376_MOESM1_ESM.docx]

**Table S1: PCR primers and their characteristics restriction enzymes**

Table S1 shows the primers spanning each of the SNP variants (rs2296651, rs61745930, and rs4646287) were designed using the NCBI Primer-BLAST software.

**Table S1: PCR primers and their characteristics restriction enzymes**

| **NTC SNPs** | **Primer** | **Primer Sequence** | **RE** |
| --- | --- | --- | --- |
| **rs2296651** | **Forward** | **5´-ATATGGCAATGAGGAGAAGC-3´** | ***HphI*** |
|  | **Reverse** | **5´-TTCCCTCTGAGTGTATGTGG-3´** |  |
| **rs61745930** | **Forward** | **5´-TATAGTCCCAGTTACTTGGGGGGCTGAG-3´** | ***BsaBI*** |
|  | **Reverse** | **5´TGTTTCCCACCTCCAATTTCTACCTGTG-3´** |  |
| **rs4646287** | **Forward** | **5’-TCTCCCCAGTTTGGAAGGATGA-3’** | ***Taq^ἀ^I*** |
|  | **Reverse** | **5’-AGAGTTTCCCAGCACCCACTCC-3’** |  |

***RE, Restriction Enzymes***

**Table S2: Hematological profile of the study participants**

Table S2 shows the measured hematological parameters among cases and control subjects

**Table S2: Hematological profile of the study participants**

| **Variables** | **Cases**  **(n=146)** | **Control**  **(n=146)** | **P-value** |
| --- | --- | --- | --- |
| **Hb (13.0 -18.0M; 12.0 -16.0F) g/dL** | 12.3(2.1) | 12.9(1.9) | **0.009** |
| **Total WBC count (4.3 - 11.5) 109/L** | 5.9(1.8) | 6.8(3.1) | **<0.001** |
| **Neutrophils (50.0 - 70.0%)** | 45.9(13.4) | 57.6(15.3) | **<0.001** |
| **Lymphocytes (20.0 - 40.0%)** | 44.1(13.0) | 32.8(14.2) | **<0.001** |
| **Monocytes (3.0 -12.0%)** | 5.8(4.8) | 6.9(2.7) | **0.013** |
| **Eosinophils (0.5 - 5.0%)** | 3.2(3.1) | 2.5(2.8) | **0.028** |
| **Basophils (0.0 -1.0%)** | 1.6(1.6) | 0.3(0.6) | **<0.001** |
| **Platelets (100-300)x109/L** | 195(53) | 222(70) | **<0.001** |
| **Hematocrit (37.0 - 54.0%)** | 36.7(6.7) | 35.4(5.9) | 0.088 |
| **RBC's (3.5-5.5)x1012/L** | 4.3(0.7) | 4.2(0.6) | 0.134 |
| **MCV (80.0 -100.0 fL)** | 83.2(11.1) | 83.9(7.5) | 0.474 |
| **MCH (27.0 -34.0 pg)** | 28.3(5.5) | 30.9(5.7) | **<0.001** |
| **MCHC (32.0 -36.0 g/dL)** | 32.9(3.2) | 36.2(3.0) | **<0.001** |
| **RDW-CV (11.0 -16.0%)** | 15.3(4.4) | 13.4(1.4) | **<0.001** |
| **RDW-SD (35.0 - 56.0 fL)** | 46.7(7.8) | 42.8(5.4) | **<0.001** |
| **PDW(9 -17)** | 14.5(3.2) | 15.9(2.3) | **<0.001** |
| **PCT (0.20 - 0.35%)** | 0.23(0.07) | 0.22(0.07) | 0.59 |
| **MPV (8.0 - 11.5fL)** | 12.2(4.3) | 9.8(1.3) | **<0.001** |

**Table S3: Antigens and host antibodies profiling of HBV positive cases**

Table S3 shows the re-categorised into HBeAg positive (active) and HBeAg negative (inactive) based on HBV antigen/antibody profiling.

**Table S3: Antigens and host antibodies profiling of HBV positive cases**

| **Antigen/antibody** | **Positive results** | **Negative results** |
| --- | --- | --- |
| **HBsAg (%)** | 146/146 (100.0%) | 0 |
| **HBsAb (%)** | 5/146 (3.4%) | 141/146 (96.6%) |
| **HBeAg (%)** | 57/146 (39.0%) | 142/146 (61.0%) |
| **HBeAb (%)** | 4/146 (5.5%) | 89/146 (94.5%) |
| **HBcAb (%)** | 139/146 (95.2%) | 7/146 (4.8%) |

**Figure S1**: Gel bands for the SNP rs2296651 obtained using the PCR-RFLP.

**Figure S1** shows the gel bands for the SNP rs2296651 obtained using the PCR-RFLP. MM= the molecular marker or ladder; NC = the negative control; C (200bp), T (120bp). Samples 51 – 60 are cases and samples 291 – 300 are controls.

**Figure S2:** Gel bands for the SNP rs61745930 obtained using the PCR-RFLP.

**Figure S2** shows the gel bands for the SNP rs61745930 obtained using the PCR-RFLP. MM= the molecular marker or ladder; NC = the negative control; C (400bp), T (250). Ca = Cases and Co= Controls. Samples 22 –25, 27, 30 and 33 are cases and samples 243, 249,252, 318, 320 – 322 are controls.

**Figure S3:** Gel bands for the SNP rs4646287 obtained using the PCR-RFLP.

**Figure S3** shows the gel bands for the SNP rs4646287 obtained using the PCR-RFLP. MM= the molecular marker or ladder; C(240bp), T (140). Samples 35 – 46 are cases; and samples 201 – 205 plus 286 - 290 are controls.
