# Supplementary figures and images for: NTCP gene polymorphisms and hepatitis B virus infection status in a Ghanaian population
Source: Virol J. 2020 Jul 3;17:91. doi: 10.1186/s12985-020-01376-0 (PMC7333392; doi:10.1186/s12985-020-01376-0)

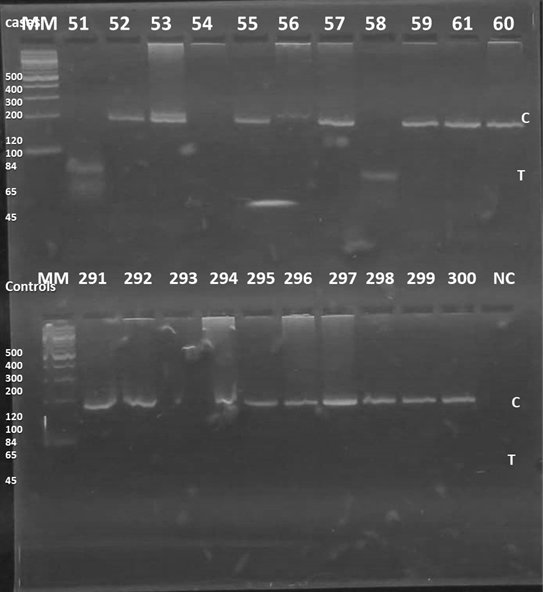

Supplement: Supplementary file 2 — Additional file 2: Figure S1. Gel bands for the SNP rs2296651 obtained using the PCR-RFLP. Figure S1 shows the gel bands for the SNP rs2296651 obtained using the PCR-RFLP. MM = the molecular marker or ladder; NC = the negative control; C (200 bp), T (120 bp). Samples 51–60 are cases and samples 291–300 are controls. [file 12985_2020_1376_MOESM2_ESM.tif]

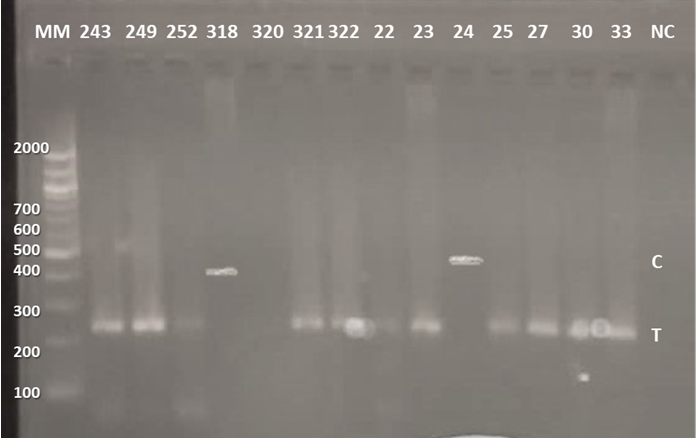

Supplement: Supplementary file 3 — Additional file 3: Figure S2. Gel bands for the SNP rs61745930 obtained using the PCR-RFLP. Figure S2 shows the gel bands for the SNP rs61745930 obtained using the PCR-RFLP. MM = the molecular marker or ladder; NC = the negative control; C (400 bp), T (250). Ca = Cases and Co = Controls. Samples 22–25, 27, 30 and 33 are cases and samples 243, 249,252, 318, 320–322 are controls. [file 12985_2020_1376_MOESM3_ESM.tif]

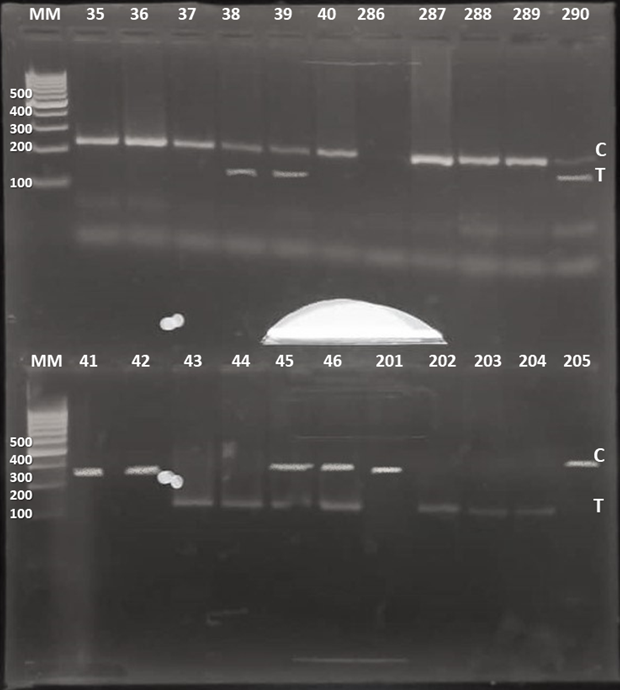

Supplement: Supplementary file 4 — Additional file 4: Figure S3. Gel bands for the SNP rs4646287 obtained using the PCR-RFLP. Figure S3 shows the gel bands for the SNP rs4646287 obtained using the PCR-RFLP. MM = the molecular marker or ladder; C (240 bp), T (140). Samples 35–46 are cases; and samples 201–205 plus 286–290 are controls. [file 12985_2020_1376_MOESM4_ESM.tif]
